# Umbilical cord mesenchymal stem cells promote the repair of trochlear groove reconstruction in dogs

**DOI:** 10.3389/fvets.2022.922390

**Published:** 2022-08-24

**Authors:** Shi He, Jun Zhang, Wojun Chen, Yanyao Yan, Yuhong Lin, Yicheng Zhang, Shirui Lei, Chuyin Huang, Shengfeng Chen, Zhisheng Chen, Canying Liu, Yinshan Bai, Huiqin Ji, Huimin Ruan, Dongsheng Li, Cailing Ye, Cuilin Wang, Xiaoshu Zhan, Bingyun Wang

**Affiliations:** ^1^School of Life Science and Engineering, Foshan University, Foshan, China; ^2^Guangdong Polytechnic of Science and Trade, Guangzhou, China; ^3^Guangdong VetCell Biological Technology Co., Ltd., Foshan, China; ^4^Department of Animal Biosciences, University of Guelph, Guelph, ON, Canada

**Keywords:** canine, mesenchymal stem cells, patellar luxation, trochlear groove reconstruction, cartilage repair

## Abstract

Trochlear groove reconstruction (TGR) is a common treatment for patellar luxation (PL) in dogs. Nevertheless, the prognosis of TGR is poor due to the cartilage damage and secondary inflammation. To study the repair effect of canine umbilical cord mesenchymal stem cells (UC–MSCs) after TGR, 10 experimental dogs were given TGR surgery and then randomized into two groups: Treatment group (1 ml suspension allogeneic UC–MSCs (10^6^ cells/kg) was injected into the cavum articulare on days 0, 7, and 14 after TGR); and the Model group (injected with 1 ml of physiological saline as negative control). The therapeutic effect of UC–MSCs was studied by blood routine examination, inflammatory factor index detection, double-blind knee score, histopathology, and computed tomography (CT) scans. The results showed that the total number of white blood cells and neutrophils in the model group were significantly higher than those in the treatment group on both 7 days and 21 days, postoperatively (*P* < 0.05); there were no significant changes in the levels of IL-6, MMP-13, and TGF-β1 between the model group and the treatment group throughout the days of testing. The double-blind knee scores of the treatment group were significantly lower than the model group on 1st, 4th, and 5th days postoperatively (*P* < 0.05). The treatment group showed low-pain sensation, stable gait, and fast recovery of muscle strength in the knee score, and the wound healing of the treatment group returned to normal on the 5th day after surgery; CT scans and gross observation showed that the cartilage growth in the treatment group was faster than that in the model group. Histological observation of cases showed that fibro chondrocytes were predominantly found in the treatment group, and the distribution of chondrocytes was uneven, while the model group showed a large number of fibrous tissue hyperplasia, fissures, and unequal matrix staining. Intra-articular injection of UC–MSCs after TGR has the effect of relieving pain and promoting the repair of bone defects, making the operative limb recover function earlier, making up for the deficiency of TGR, and improving the effect of PL treatment. Future studies should furthermore explore the dose and frequency of therapy based on the multiple advantages of UC–MSCs and the mechanism of cartilage repair in dogs.

## Introduction

The patella is located in the femoral groove and protects the knee joint under normal physiological conditions. Patellar luxation (PL) is a clinical term used when the patella is dislocated from the trochlear groove, which is one of the most common orthopedic diseases in both large and small dogs. Among PLs, medial patellar luxation (MPL) has higher prevalence compared with that of the lateral patellar luxation (LPL) (1). LPL mostly occurs in large dogs; however, the occurrence of LPL is not related to the bodyweight of the dog (2). Several breed predispositions have been reported, including Pomeranians, which have the highest incidence at 41.2% (3, 4). Congenital genetics and acquired external forces are the two major factors that may cause the PL (5). Congenital genetic factors refer to congenital abnormal development, while acquired external factors including trauma joint capsule and fascia tear or stretch that cause patella instability. The Roush patellar luxation grade is used clinically to diagnose PL in which there are four grades in total, with higher grades being more severe and may result in severe muscle atrophy that requires early intervention (6). The debate has long prevailed as to whether surgery should be used to treat the disease. Some doctors believe that surgery should be performed at all levels of PL because of the potential risks of greater joint damage, such as arthritis and bone deformation. However, some doctors believe that surgery should only be conducted if the dog is lame. Currently, trochlear groove reconstruction (TGR) is the most commonly used surgery for the treatment of PL, as it can fix the patella to move within the chute and ensure that the midpoint of femur, patella, and tibial tuberosity are in a straight line and in the center of the knee joint (7, 8). TGR eliminates muscle atrophy, pain, and limp caused by PL. However, this surgery can also lead to cartilage damage, chronic arthritis, and other complications, which is detrimental to the postoperative recovery of the affected dogs. Therefore, prevention of postoperative inflammation and repair bone defects is critical for the repair of TGR in patellar dislocation dogs.

Mesenchymal stem cells (MSCs) are cells derived from mesoderm, which have the potential of self-renewal and multidirectional differentiation, and can be isolated from a variety of tissues and organs including bone marrow, fat, placenta, and umbilical cord (9). It is well established that MSCs have great ability to promote tissue repair, inhibit inflammation, and reconstruct body immunity (10, 11). Because of these characteristics, veterinary researchers focused on the therapeutic application of dogs and successfully isolated MSCs from different tissues, such as dental pulp (12), kidney (13), bone marrow (14), umbilical cord (12, 15), and adipose tissue (13, 16). Compared with other mesenchymal stem cells, canine UC–MSCs have the advantages of homing, low immunogenicity, no tumorigenicity, and convenient sample collection (16, 17). Moreover, canine UC–MSCs have been applied to the therapy of osteoarthritis (18) and bone defects after dental implantation (19) in which the results showed positive effects of UC–MSCs on tissue recovery. However, the application and effects of UC–MSCs in the postoperative TGR repair still remains largely unknown.

In this study, we aimed to investigate the function of UC–MSCs in the repair of TGR by injecting suspension allogeneic UC–MSCs into the cavum articulare of dogs after TGR modeling. The bone repair status of TGR surgery was assessed by four aspects: hematology, behavior, imaging, and pathology. This study might provide a new way to improve the shortcomings of TGR and provide an experimental basis for improving the therapeutic effect of PL.

## Materials and methods

### Obtain of canine UC–MSCs

The UC–MSCs used in this experiment were provided by the Guangdong Vet Cell Biological Technology Co., Ltd. UC–MSCs were isolated, purified, and characterized as described previously (20). In brief, the umbilical cord tissue was obtained from the postpartum female canine then washed with PBS containing penicillin streptomycin. The umbilical cord tissue was then shredded and detached from each other by treatment with collagenase type I for 6 h in a 37°C water bath. Cell clumps were filtered through a 100-mesh cell strain. Filtration was then collected and centrifuged at 1,000 g for 5 min. Cell pellet was re-suspended in the MSC complete medium which consists of 89% DMEM medium, 10% fetal bovine serum, and 1% Pen-Strep. Cells were then transferred into cell culture dishes and cultured in a 37°C incubator with 100% humidity and 5% CO_2_. The characteristics of UC–MSCs were assessed when confluent. To make UC–MSC injection solution, cells were detached from culture dishes by treatment with EDTA–Trypsin and then washed twice with PBS. After cell counting, cells were then resuspended in saline solution for injection.

### Animals

In total, 10 healthy poodles ranging from 12 to 18 months old and weighing from 4–6 kg were selected. Animals were randomly divided into two groups: model group and treatment group (each group has five dogs, half male and half female). They were housed in individual cages and fed a standard diet with free access to drinking water. All the studies were approved by the Animal Ethics Committee of Foshan University and conducted in accordance with the ethical standards of university. The project identification code is FOSU2022001.

### Surgical procedures of the TGR model

All the dogs were anesthetized with dexmedetomidine and sutein, and then anesthetized with isoflurane. The deep fascia and joint capsule were incised through a parallel incision centered on the patella along the straight patellar ligament on the skin of the lateral sulcus of the anesthetized right hind limb. The straight patellar ligament and patella were turned inward to completely expose the femoral trochlea. A scalpel was used to cut two parallel lines on the trochlear ridge, and then a bone saw was used to deepen the rectangular tangent line to ensure that all the cartilage reaches the subchondral bone. The cartilage flap was separated with an osteotome (only the hyaline cartilage part was retained), and the trochlear groove was deepened to spongy bone with a scalprum. The cartilage flap was trimmed and returned to its original position. The knee joint was flexed and extended to determine normal movement and friction after the patellar ligament and patella were put into the trochlea. After surgery, synovium, fasciae, and skin were sutured by *2-0 PGS*.

### Postsurgical treatment

Dogs were fitted with collars to prevent licking the bite wounds and oral Trolevis (cefalexin) at 150 mg/d for 5 days; iodophor was used to disinfect the wound twice a day, and stitches were removed 7–10 days after surgery. On days 0, 7, and 14 after TGR surgery, dogs in the treatment groups were injected with 1 ml suspension allogeneic UC–MSCs (10^6^ cells/kg) into the cavum articulare and 1 ml physiological saline solution was injected into the cavum articulare of the model group. To better operate the injection, the dog's hind right leg was bent at 90° to shift the patella up. The injection position was between the straight patellar ligament and the lateral patellar retinaculum (the lower concave can be touched), and the injection can be carried out when the needle was inserted into the cavum articulare. Postoperative care and rehabilitation exercises were consistent with each dog.

### Blood routine examination and enzyme-linked immunosorbent assay

On days 3, 7, 11, 15, and 21 after surgery, 2 ml blood was collected from the cephalic vein of the forelimb or saphenous vein of the hindlimb of each dog in an EDTA tube. In total, 1 ml blood was taken for routine blood test, while another 1 ml was centrifuged at 2,500 r/min for 10 min to isolate plasma and stored at −80°C. The concentration of transforming growth factor-β1 (TGF-β1), interleukin-6 (IL-6), and matrix metallopeptidase-13 (MMP-13) in plasma were determined using the ELISA kits (Jiangsu Meimian Industrial Co., Ltd, Jiangsu, China).

### Canine knee joint recovery score

On day 1 to day 7 after surgery, dogs in both the model group and treatment group were scored for their behavior and postoperative knee joint recovery according to blind evaluation method from wound, pain, gait analysis, step up and down experiment, and range of motion listed in Table 1.

**Table 1:** Canine knee joint recovery score table in total.

**Indicators**	**Score**	**Criteria**
Wound recovery	0	normal
	1	slight swelling
	2	slight swelling and bleeding
	3	Exudation, hemorrhage
Pain response	0	no pain response
	1	mild pain during physical stimulation
	2	occasional pain while walking
	3	pain during human activities
	4	pain when walking
	5	pain
Gait analysis	0	normal
	1	abnormal running and normal walking
	2	abnormal running and walking
	3	resisting the movement
	4	standing intolerance
Up and down	0	normal
	1	upstairs normal, downstairs need traction
	2	need traction
	3	need traction upstairs, downstairs difficult
	4	difficult
Strength	0	totally resistant to resistance
	1	partial resistance
	2	normal joint motion
	3	abnormal joint motion

### Observation of the growth status of the patella and cartilage by CT scan

Computed tomography scans of the knee joint were performed on days 9, 23, 37, 51, and 65 postoperatively. The position was interpreted as a coronal plane, showing the plane with the maximum width of the patella, and the femoral trochlear cartilage and bone surface below the patella were observed.

### Histological examination

At 82 days postoperatively, four dogs were randomly selected for gross and histopathological observations. Euthanasia was performed by mixing propofol with potassium chloride (0.8 ml/kg of propofol, followed by 10 ml of potassium chloride, intravenously). The synovium, articular capsule, and cartilage of dogs were observed in general, to observe whether there was adhesion or hyperplasia and to note the color, transparency, and smoothness of the neogenetic cartilage at the junction of cartilage flap and cartilage.

The cartilage tissue from the damaged part of TRG was collected and fixed with 4% paraformaldehyde for 48 h. After fixation, tissues were decalcified and dehydrated. When the pin could penetrate the specimen without resistance, the specimen size was cut, paraffin embedding, sectioning, hematoxylin–eosin (H&E) staining, and Masson staining were carried out, and the cell morphology, distribution, and matrix staining of the cartilage tissue under sectioning were observed under the microscope.

### Statistical analysis

Statistical analysis of all the data was performed using PRISM7.0 software. The results were presented as mean ± standard error ($\bar{x}$ ± SEM). Statistical significance was measured with *t*-test. Differences at *P* < 0.05 were considered significant.

## Results

### Changes in WBC and neu after surgery

At each of the five time points (Table 2), the total number of both white blood cells and neutrophils were within the normal range in both the groups. The total number of both white blood cells and neutrophils showed no significant difference between the model group and the treatment group on the 3rd, 11th, and 15th days after the TGR. However, compared with the model group, the total number of both white blood cells and neutrophils in the treatment group were significantly lower than the model group on day 7 and day 21 postoperatively (*P* < 0.05). Specifically, on day 21 postoperatively, the total number of neutrophils had a highly significant difference within the two groups, in which the treatment group was lower than the model group (*P* < 0.01).

**Table 2:** Blood routine examination results (x ± SEM, *n* = 5).

**Group**	**Indicators**	**3d**	**7d**	**11d**	**15d**	**21d**	**Normal range**
WBC^a^	Treatment group	14.38 ± 2.16	9.16 ± 1.46^*^	11.25 ± 1.82	12.33 ± 1.87	9.09 ± 1.33^*^	6.00–17.00
(× 10^9^/L)	Model group	15.36 ± 1.16	14.22 ± 0.64	14.89 ± 1.13	13.17 ± 0.95	13.80 ± 1.07	6.00–17.00
Neu^b^	Treatment group	9.17 ± 1.34	5.85 ± 1.01^*^	6.98 ± 1.24	7.91 ± 1.31	5.26 ± 0.68*^*^	4.00–12.60
(×10^9^/L)	Model group	9.89 ± 0.64	9.32 ± 0.53	9.76 ± 0.75	8.25 ± 0.47	8.59 ± 0.68	4.00–12.60

a WBC represents the number of white blood cells.

b Neu represents the number of neutrophils.

* Significance was measured using a t-test. Values are presented by means ± standard error for n = 5 dogs. Astricts signify that the cell number is significantly different in comparison to the model group at the same time point (*p < 0.05 and **p < 0.01).

### Plasma inflammatory factor detection results

The ELASE results (Table 3) showed that there was no significant difference in the levels of IL-6, MMP-13, and TGF-β1 between the model group and the treatment group on the days of testing.

**Table 3:** The results of blood content of cytokines (x ± SEM, *n* = 5).

**Indicators unit**	**Group**	**7d**	**14d**	**18d**	**28d**
TGF-β1 pg/mL	Model group	225.20 ± 13.63	226.80 ± 6.57	200.50 ± 12.60	234.00 ± 6.42
	Treatment group	242.50 ± 3.91	235.10 ± 15.12	212.10 ± 11.95	230.70 ± 9.70
IL-6 pg/mL	Model group	279.80 ± 15.99	281.80 ± 18.03	278.30 ± 10.95	309.40 ± 14.55
	Treatment group	251.80 ± 5.14	257.00 ± 10.55	253.10 ± 12.05	283.40 ± 10.20
MMP-13 pg/mL	Model group	274.00 ± 12.61	253.40 ± 9.99	239.90 ± 6.04	270.00 ± 7.11
	Treatment group	253.00 ± 4.52	230.00 ± 3.85	225.00 ± 7.07	270.90 ± 9.92

### Behavioral knee score results

The total score results in Table 4 showed that there was no significant difference between the model group and the treatment group on the 2nd, 3rd, 6th, and 7th days after the TGR. However, compared with the model group, the total score of the treatment group was significantly lower than the model group after 4 days and 5 days postoperatively (*P* < 0.05). Specifically, the total score had a very significant difference in the two groups on the 1st day postoperatively (*P* < 0.01). A lower score indicates a better recovery.

**Table 4:** The results of knee joint totally score (x ± SEM, *n* = 5).

**Group**	**1d**	**2d**	**3d**	**4d**	**5d**	**6d**	**7d**
Treatment group	14.60 ± 0.40**	14.00 ± 0.77	12.80 ± 1.28	9.60 ± 1.08^*^	8.60 ± 1.21^*^	8.60 ± 1.78	6.20 ± 1.74
Model group	17.20 ± 0.37	15.80 ± 1.24	16.00 ± 0.77	14.00 ± 1.18	12.60 ± 1.03	12.40 ± 1.54	11.40 ± 1.53

* Significance was measured using a t-test. Values are presented by means± standard error for n = 5 dogs. Astricts signify that the total knee joint recovery score is significantly different in comparison to the model group at the same time point (*p < 0.05 and **p < 0.01).

The results in Table 5 showed the wound recovery score, with dogs in the treatment group returning to normal score 0 on day 5; the scores of pain response in the treatment group were lower than the model group on day 4 (*P* < 0.05); the scores of strength in the treatment group were lower than the model group on days 4 and 5 (*P* < 0.01). During the seven-day observation period, the other indicators were not scored as “0” except wound recovery.

**Table 5:** The results of knee joint score ($\bar{x}$ ± SEM, *n* = 5).

**Indicators**	**1d**	**2d**	**3d**	**4d**	**5d**	**6d**	**7d**
**Wound recovery**							
Model group	1.60 ± 0.24	1.40 ± 0.24	0.60 ± 0.24	0.40 ± 0.40	0.20 ± 0.20	0.20 ± 0.20	0.20 ± 0.20
Treatment group	1.20 ± 0.20	1.20 ± 0.20	0.80 ± 0.37	0.40 ± 0.24	0.00 ± 0.00	0.00 ± 0.00	0.00 ± 0.00
**Pain response**							
Model group	5.00 ± 0.00	4.20 ± 0.80	4.80 ± 0.20	4.20 ± 0.58^*^	3.60 ± 0.60^*^	3.40 ± 0.75	3.20 ± 0.80^*^
Treatment group	4.00 ± 0.00	4.20 ± 0.20	3.60 ± 0.51	2.20 ± 0.58	1.80 ± 0.37	2.20 ± 0.80	0.60 ± 0.40
**Gait analysis**							
Model group	3.80 ± 0.20	3.60 ± 0.40	3.80 ± 0.20	3.40 ± 0.40^*^	3.60 ± 0.24^*^	3.00 ± 0.45	2.60 ± 0.40
Treatment group	3.20 ± 0.20	2.40 ± 0.40	2.80 ± 0.37	1.80 ± 0.37	2.00 ± 0.63	1.80 ± 0.58	1.40 ± 0.75
**Up and down**							
Model group	4.00 ± 0.00	4.00 ± 0.00	4.00 ± 0.00	4.00 ± 0.00	3.40 ± 0.24	4.00 ± 0.00^*^	3.80 ± 0.20
Treatment group	4.00 ± 0.00	4.00 ± 0.00	4.00 ± 0.00	4.00 ± 0.00	3.60 ± 0.24	3.40 ± 0.24	3.20 ± 0.37
**Strength**							
Model group	2.80 ± 0.20	2.60 ± 0.24	2.80 ± 0.20*^*^	2.00 ± 0.00*^*^	1.80 ± 0.20	1.80 ± 0.37	1.60 ± 0.24
Treatment group	2.20 ± 0.20	2.20 ± 0.20	1.60 ± 0.24	1.20 ± 0.20	1.20 ± 0.20	1.20 ± 0.20	1.00 ± 0.32

* Significance was measured using a t-test. Values are presented by means± standard error for n = 5 dogs. Astricts signify that the knee joint recovery score in each item is significantly different in comparison to the model group at the same time point (*p < 0.05 and **p < 0.01).

### Computed tompography detection results

**Figure 1:**
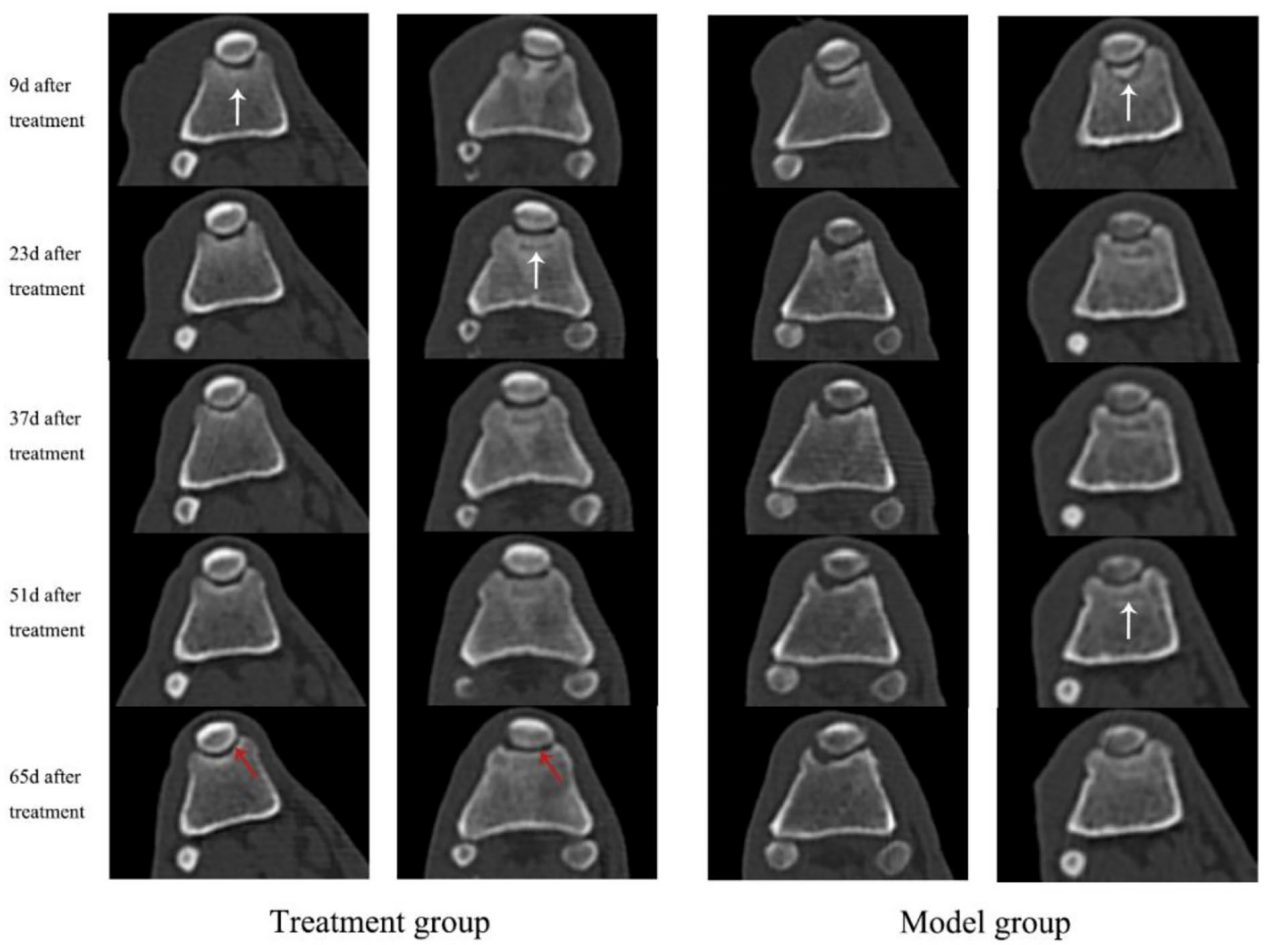
CT scan images of knee-joint from both treatment group and model group in days 9, 23, 37, 51, and 65, respectively. The white arrows indicate the interfaces and periosteum of the trochlea, while the red arrows indicate the cartilage tissue of trochlear groove.

A healthy trochlear groove is characterized by high echo, clear shape, continuous margin, and uniform bone density. As shown in Figure 1, on day 9 after surgery, the CT images of the knee-joint in both the treatment group and model group showed irregular margins of the trochlea, incomplete shape, and periosteum. There was a bone defect between the cartilage flap and the bone tissue polished by the bone saw (white arrow). In the treatment group, the cartilage tissue of the trochlear groove appeared hyperechoic with continuous and neat edges, and the bone contour was basically restored on the 65th day (red arrow). The cartilage defect gradually increased in density and formed connections and the earliest time to return to normal was on the 37th day. During 9–65 d, the model group showed an obvious interfacial gap between cartilage flap and damaged bone tissue (white arrow), and an uneven profile of trochlear groove. In addition, some experimental dogs showed hyperosteogeny and incomplete periosteum. Bone contour and bone mineral density were greater in the treatment group compared with the model group.

### Recovery of the operative incision observation

**Figure 2:**
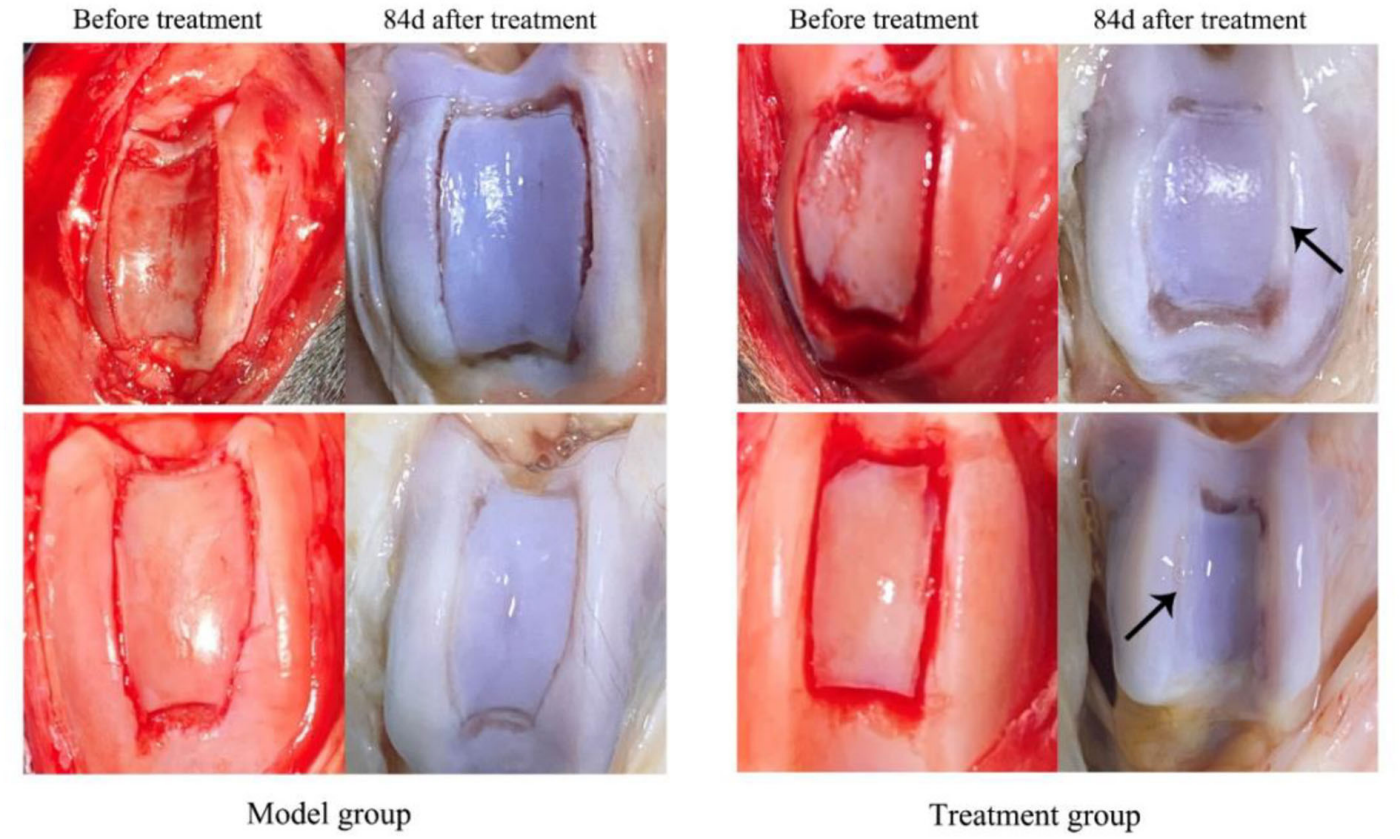
The appearance of knee joint after treatment for 84 d in both model group and treatment group. The black arrows indicate the sugery sites and cartilage tissue regenerated in the trochlear groove.

Cartilage flap and bone tissue were damaged and the ridge of the trochlea was cracked in both groups of dogs after TGR, as shown in Figure 2. The wound skin of the two groups healed well without adhesion and hyperplasia in subcutaneous tissue after 84 days postoperatively. The joint capsule structure was complete with no damage and thickening. The joint fluid was clear with no blood or turbidity. After three doses of treatments with UC–MSCs (84 days after surgery), new white cartilage tissue appeared at the bone defect of trochlea in the treatment group, forming connections with surrounding cartilage tissue (black arrow). The degree of cartilage defect in the model group was similar to that on the day of surgery without obvious new cartilage tissue.

### Histopathological observation results

**Figure 3:**
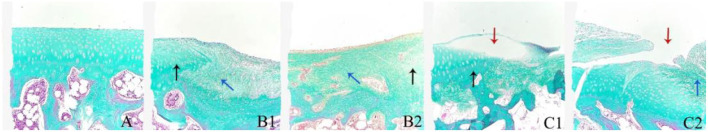
The representative histological sections of the knee joint cartilage in day 84 in the treatment group **(B1,B2)**, model group **(C1,C2)**, and normal cartilage for reference **(A)**. Masson staining, 100×.

As shown in Figure 3A, in the healthy cartilage section, the chondrocytes were arranged neatly, the cartilage matrix was uniformly stained, and the perichondrium was intact and continuous. The cartilage matrix was not uniformly stained (blue arrow) and chondrocyte defects were observed in both treatment and model groups. The sections of the treatment group (Figure 3B) showed cartilage defects and fibrous hyperplasia. There were neogenetic chondrocytes and cartilage collagen (black arrow), and the perichondrium was continuous without fissure. In the model group (Figure 3C), cartilage was absent at the damaged site accompanied by fibrous tissue hyperplasia, with a few neogenetic chondrocytes, but chondrocytes were disordered. The perichondrium was fissured, unevenly dense, and vacuolated (red arrow).

**Figure 4:**
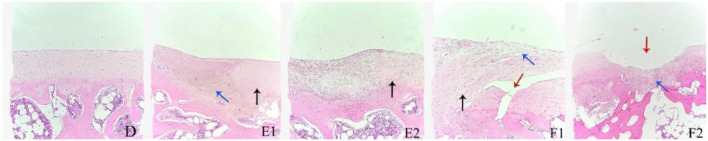
The representative histological sections of the knee joint cartilage in day 84 in the treatment group **(E1,E2)**, model group **(F1,F2)**, and normal cartilage for reference **(D)**. H&E staining, 100×.

In the histological section of normal cartilage, smooth and continuous stratum fibrosum were shown distinctly with orderly arrangement of chondrocytes, zone of calcifying cartilage, and subchondral bone (Figure 4D). As shown in Figures 4E,F, both the treatment group and the model group showed uneven staining of the cartilage matrix and uneven periosteum profile with fibrous hyperplasia (blue arrow). In the treatment group (Figure 4F), chondrocytes were disordered and the boundary of the zone of cartilage was not obvious. Nonetheless, a large number of neogentic chondrocytes (black arrow) were observed, and the perichondrium was continuously free of cracks. The cartilage tissue of the model group (Figure 4F) was damaged and lost, and the cartilage fibrous tissue was hyperplasia. The surface and deep layers of cartilage are damaged and structurally disorganized with cracks (red arrows). Abnormal chondrocytes were observed in some areas, and a few neoregentic chondrocytes were observed.

## Discussion

There are plenty of factors that may cause PL in dogs which include coxa vara, femoral torsion, edial rotation of the tibia, hypoplastic medial trochlear ridge, excessive distal femoral varus, cranial cruciate ligament (CrCL), and acquired external factors (5). In the past, clinical researchers have focused on technical improvements in the treatment of PL, such as trochlear wedge recession, trochlear block recession, and semi-cylindrical trochleoplasty. In order to solve the defect of postoperative injury, total knee replacement came into being. Even with the advent of new techniques, TGR remains the most basic surgical procedure. Clinically, most PL can be solved by combining soft tissue and bone tissue reconstruction (21). Although TGR has been continuously improved to reduce the degree of cartilage flap damage, its disadvantages of bone tissue damage, postoperative inflammation, and complications still exist. Cartilage, which has no blood supply and relies on joint fluid for nourishment, is difficult to repair once damaged. In order to improve the quality of postoperative recovery in animals, the combined application of TGR and intra-articular injection UC–MSCs has great potential.

Many studies found that MSCs have the capability of regenerative repair, anti-inflammatory, and immune remodeling, and have been widely used to treat tissue and organ injury, inflammation, and autoimmune diseases (22, 23). However, its mechanism of action is still unclear.

Current studies have found that MSCs directly play a minimal role in tissue repair through cell differentiation (24, 25), while paracrine (cytokines, growth factors, and exosomes) (26) and immune regulatory pathways (27) are the main mechanisms of their *in vivo* treatment. In this study, IL-6, TGF-β1, and MMP-13 were selected for observation. The metabolic activities of chondrocytes are mainly affected by the inflammatory cytokines and growth factors, and IL-6 can induce chondrocytes to secrete MMP-13 to promote apoptosis (28). Liu et al. found that UC–MSCs can inhibit the production of inflammatory cytokines and improve osteogenic differentiation in collagen-induced arthritis (CIA) (29). TGF-β family plays an important role in the process of cartilage and bone formation. TGF-β1 can stimulate the proliferation of MSCs and promote their chondrogenic differentiation (30). Furthermore, MSCs can secrete TGF-β1 to promote the expression of glycosaminoglycan, SOX-9, and peroxisome proliferator-activated receptor gama coactivator 1 alpha (PGC-1α), and inhibit the expression of MMP-3 and MMP-13, which destroy cartilage regeneration (31). Rostiny's study showed that transplantation of UC–MSCs recruited more MSCs to the injury site for proliferation and differentiation by up-regulating TGF-β1 expression and accelerating cartilage generation (32). Barry and Murphy suggested that endogenous MSCs reduce inflammation principally by acting as reservoirs for cell repair or as sentinels for immune regulation and that paracrine of MSCs may be more important than differentiation (33). Zhang et al. confirmed the effect of MSC–Exo on cartilage repair (34). It is reported that MSC–Exo also plays a role in cartilage protection by inhibiting the expression of MMP-13 (35, 36). Meng et al. found that miR-320 in MSC–Exo alleviated inflammatory injury of chondrocytes by downregulating the expression of MMP-13 (37). In this study, there were no statistically significant differences in IL-6, TGF-β1, and MMP-13 between the two groups. This might be because the cytokine secretion regulation networks and signaling pathways *in vivo* are more complicated than that of the *in vitro* studies. There might be delicate crosstalks between tissues to maintain the body homeodynamic during surgery recovery which leads to the non-significant changes of the tested cytokines observed in our study. Therefore, the authors believe that the underlying mechanism of UC–MSCs in the treatment of bone repair in dogs needs to be further verified.

Trochlear groove reconstruction requires the destruction of skin, subcutaneous tissue, joint capsule, cartilage, and part of the bone tissue. Postoperative inflammation will lead to swelling and pain in the operative area, which is not conducive to the recovery of affected dogs. The inflammatory reaction is manifested as the increase of the total number of white blood cells. The results of this study showed that the total number of white blood cells and neutrophils in the model group were significantly higher compared to the treatment group on 7 and 21 days postoperatively (*P* < 0.05).

After TGR, the trochlear groove will become rough, and when the patella slips, secondary cartilage wear occurs, resulting in reduced range of motion, which affects the recovery of the postoperative knee function. The results of this experiment showed that the treatment group showed low-pain sensation, stable gait, and fast recovery of muscle strength in the knee score, and the wound healing of the treatment group returned to normal on the 5th day after surgery. The pain response, gait analysis, and muscle strength of the treatment group and the model group could not return to normal values during the observation period, but the treatment group showed a trend of faster recovery since the 4th day, and dogs in the treatment group showed more willingness to walk and run on the operation legs. Although the treatment group could not recover completely within 7 days of the observation period, it suggested that UC–MSCs treatment could reduce the postoperative pain response and promote the recovery of knee function.

Imaging is a common way to evaluate the complications of treatment for PL. CT scans can observe whether the patella dislocated again and the growth of cartilage and bone tissue in the trochlear groove. The CT scans results of this experiment showed that the bone density of the bone defect in the treatment group was higher than the model group. The cartilage tissue of trochlear groove appeared hyperechoic with continuous and neat edges. The damaged cartilage tissue of the treatment group returned to normal as early as 37 days postoperatively, while the model group did not return to a normal cartilage state until the end of the experiment (12 w). Histopathological analysis of this study revealed that there was new cartilage tissue in the trochlea of the treatment group on the 84th day postoperatively, and it was connected with the surrounding cartilage tissue. Periosteum is continuous without fissure. The cartilage tissue of the model group was thin and accompanied by fibrous hyperplasia. These results suggest that the regeneration of cartilage and bone tissue in the treatment group was better than that in the model group. Zhang et al. used hUC–MSCs to treat the joint injury caused by MIA in mice, showing that the cartilage fissure disappeared and the thickness of chondrocytes and cartilage increased (38). An et al. found that UC–MSCs, however, slightly aggravated arthritis in CIA mice after treatment for 84 days when injected three times a week for 3 weeks at a high dose of 5 × 10^6^ cells and frequently (39). The therapeutic effect may depend on the number of cells and the frequency of injections. In this study, multiple injection therapy was used, with an interval of 7 days for each injection and three times as a course of treatment. The concentration of UC–MSCs was 1 × 10^6^/kg, which provides a fundamental basis for the future clinical application of MSCs.

## Conclusion

Intra-articular injection of UC–MSCs after TGR has the effect of relieving pain and promoting the repair of bone defects, making the operative limb recover function earlier, making up for the deficiency of TGR, and improving the effect of PL treatment. Future studies should further explore the dose and frequency of therapy based on the multiple advantages of UC–MSCs and the mechanism of cartilage repair in dogs.

## Data availability statement

The raw data supporting the conclusions of this article will be made available by the authors, without undue reservation.

## Ethics statement

The animal study was reviewed and approved by Animal Ethics Committee of Foshan University.

## Author contributions

SH contributed to the experimental design, data collection, data statistical analysis, and manuscript writing. JZ performed the experimental design, experiment preparation, animal modeling, and data statistical analysis. WC, YY, and YL participated in the execution of animal trial, data collection, and animal care facilities maintenance. SC, ZC, CL, YB, and HJ contributed to the experimental design and supervised the execution of animal trial. HR, DL, CY, and CW participated in the stem cells preparation and quality control. XZ and BW contributed significantly to the experimental design, animal trial supervision, data statistical analysis, and manuscript editing and discussion. All authors contributed to the article and approved the submitted version.

## Funding

This work was supported by the Natural Science Foundation of Guangdong Province, China (2020A1515011110) and Guangdong Provincial Engineering Research Center for Animal Stem Cells of Ordinary Universities (2021GCZX006).

## Conflict of interest

Authors HR, DL, CY, and CW were employed by the Guangdong VetCell Biological Technology Co., Ltd. The remaining authors declare that the research was conducted in the absence of any commercial or financial relationships that could be construed as a potential conflict of interest.

## Publisher's note

All claims expressed in this article are solely those of the authors and do not necessarily represent those of their affiliated organizations, or those of the publisher, the editors and the reviewers. Any product that may be evaluated in this article, or claim that may be made by its manufacturer, is not guaranteed or endorsed by the publisher.
